# Psychometric assessment of the Competence in Research Utilisation instrument in a sample of graduating nursing students in three European countries

**DOI:** 10.1177/17449871261463995

**Published:** 2026-08-23

**Authors:** Asta Heikkilä, Minna Stolt, Maija Hupli, Jouko Katajisto, Boris Miha Kaučič, Katarzyna Kwiecień-Jaguś, Helena Leino-Kilpi, Leena Salminen, Riitta Suhonen

**Affiliations:** Clinical Lecturer, Department of Nursing Science, University of Turku, Turku, Finland; Professor, Department of Nursing Science, University of Turku, Turku, Finland; Satakunta Central Hospital, The Wellbeing Services County of Satakunta, Pori, Finland; Department of Nursing Science, University of Turku, Turku, Finland; Lecturer, Department of Mathematics and Statistics, University of Turku, Turku, Finland; Associate Professor, Institute for Training, Work and Care Dr. Marjan Borštnar Dornava, Dornava, Slovenia; Faculty ECM, Nursing Department, Alma Mater Europaea University, Maribor, Slovenia; Assistant Professor, Department of Anesthesiology Nursing & Intensive Care, Faculty of Health Sciences, Medical University of Gdansk, Gdansk, Poland; Professor (emerita), Researcher, Department of Nursing Science, University of Turku, Turku, Finland; Turku University Hospital, The Wellbeing Services County of Southwest Finland, Turku, Finland; Professor (emerita), Researcher, Department of Nursing Science, University of Turku, Turku, Finland; Turku University Hospital, The Wellbeing Services County of Southwest Finland, Turku, Finland; Professor, Director of Nursing, Department of Nursing Science, University of Turku, Turku, Finland; Turku University Hospital, The Wellbeing Services County of Southwest Finland, Turku, Finland

**Keywords:** assessment, evidence-based practice, instrument, nursing education, research utilisation, psychometric testing

## Abstract

**Background:**

Evidence-based practice is an internationally accepted healthcare standard grounded in research utilisation. Validated instruments allow evaluation of nursing education against European Union competence requirements.

**Aim:**

To assess the psychometric properties of the Competence in Research Utilisation (CompRU) instrument among graduating nursing students using data from three European countries.

**Methods:**

Data were collected from graduating nursing students (*n* = 648) in Finland, Poland and Slovenia between 2012 and 2017. The CompRU instrument comprises a knowledge test and self-reported scales assessing skills and attitudes. Psychometric properties were assessed using Cronbach’s coefficient alpha, item-to-total correlation, Rasch analysis and confirmatory factor analysis.

**Results:**

Internal consistency reliability was acceptable in all sections, despite two categories in the Knowledge section showing lower consistency. Rasch analysis supported the internal scale validity of the Knowledge section in terms of item infit and item separation. Confirmatory factor analysis confirmed the structure (Skills and Attitudes sections) and strength of the hypothetical model.

**Conclusion:**

The CompRU instrument is a promising tool for measuring nursing students’ research utilisation competence at graduation. Based on both classical and modern test theory approaches, the instrument demonstrates evidence of acceptable psychometric properties, although some items in the Knowledge section could benefit from future revisions.

## Introduction

This study focuses on the psychometric testing of the Competence in Research Utilisation (CompRU) instrument (© Heikkilä, 2005) in a sample of nursing graduates in three European countries. As research utilisation (RU) is a fundamental component of evidence-based practice (Belita et al., 2020; Florczak, 2016), assessing competence in RU is essential. Evidence-based practice is a problem-solving approach that combines the best research knowledge with clinical expertise and patient preferences to promote safe patient care, improve quality of care and reduce the risk of adverse events (Ferreira et al., 2021; Unal and Teskereci, 2022). This study focuses on RU to highlight its role as a key driver of evidence-based practice, which is the ultimate goal of RU. Deficiencies in RU competence among nurses constitute a significant obstacle to evidence-based practice (Ferreira et al., 2021; Hines et al., 2022).

Despite harmonisation efforts of nursing education in Europe (Directive 2005/36/EC; Directive 2013/55/EU), the integration of evidence-based practice and RU competencies varies across undergraduate nursing programmes (Dolezel et al., 2021; Skela-Savič et al., 2020), even though evidence-based practice is defined as a core competency of nurse educators worldwide (World Health Organization, 2016). Thus, prioritising the teaching of both evidence-based practice and RU in undergraduate nursing education is essential (Patelarou et al., 2021). Assessment is needed to determine whether RU competences are reached in nursing education in Europe, as required in the European Union directives and to provide knowledge for the further development of nursing education. As graduating nursing students are transitioning from education to professional practice, evaluating their RU competencies also provides insight into how well nursing education prepares students to meet the competence demands of professional working life. However, no psychometrically validated instrument currently exists to comprehensively assess graduating nursing students’ RU competencies. Therefore, the psychometric properties of the CompRU instrument should be evaluated.

## Background

Four types of RU were verified as early as the 1990s (Estabrooks, 1999): (a) instrumental use, where research knowledge is applied concretely to nursing practice and decision-making; (b) conceptual use, where research findings enhance an individual’s understanding; (c) persuasive or symbolic use, where research knowledge is employed to convince others to change, such as through policy-making; and (d) general use, which involves the use of research knowledge in any form within clinical practice (Estabrooks, 1999; Estabrooks et al., 2011). However, identifying clear demarcation between the different types of RU can be difficult (Strandberg et al., 2014), and RU can occur in related forms, especially regarding instrumental and conceptual use (Estabrooks et al., 2011). Some researchers suggest that RU can be conceptualised as a gradual or a continuous process (Estabrooks et al., 2011; Strandberg et al., 2014). In this study, RU is characterised as the process of identifying research needs and acquiring, critically reading and applying research (Florczak, 2016; Heikkilä, 2005), encompassing the aforementioned theoretical structure.

There is no universally accepted definition of competence in nursing practice, though most descriptions highlight knowledge, skills and attitudes required to fulfil professional responsibilities (Mrayyan et al., 2023). In this study, competence in RU comprises knowledge and skills in RU and attitudes to RU (Heikkilä, 2005). Knowledge consists of facts, concepts, practices and theories, while skills refer to activities involving the application of knowledge (Vitello et al., 2021). Knowledge and skills in research acquisition require the identification of research needs, familiarity with relevant research databases and an effective search strategy, including the use of specific keywords and filters (Costa et al., 2025; Heikkilä, 2005). Critical reading is the ability to systematically analyse research and to distinguish between reliable and unreliable information. It requires recognising the different stages of the research process, understanding research concepts, language and statistics and assessing the reliability and validity of the research and the usefulness of the results (Heikkilä, 2005; Hines et al., 2022). Applying research may involve integrating research findings into a nurse’s continuous professional development or applying summarised research findings and nursing guidelines to patient care in a clinical and shared decision-making process that combines the best research knowledge and other elements of evidence-based practice (Estabrooks et al., 2011; Heikkilä, 2005; Unal and Teskereci, 2022). Attitudes reflect individual’s perceptions of what is important, relevant and appropriate (Price, 2015).

Many instruments have been developed to measure competence in nursing research (Qiu et al., 2019; Xia et al., 2023) as well as the attitudes and beliefs, self-efficacy, knowledge and skills and implementation of evidence-based practice (Halm, 2024). However, these instruments non-specifically and only partially cover issues relating to RU competence. Typically, only one competence area – attitudes, knowledge or skills – is evaluated at a time (Belita et al., 2020; Skela-Savič et al., 2020), mostly relying on self-report measures (Halm, 2024). Given that self-assessment accuracy is often low (González-Betancor et al., 2019), objective measures, such as a knowledge test, should also be incorporated (Hagedorn Wonder and Spurlock, 2020), as knowledge is an essential part of RU competence assessment (Hu et al., 2024).

Most of the instruments assessing attitudes, knowledge or skills in research or evidence-based practice have been developed for graduated nurses (Belita et al., 2020; Halm, 2024; Xia et al., 2023); only a few have been specifically designed for implementation in undergraduate nursing education (Halm, 2024; Qiu et al., 2019; Ruzafa-Martinez et al., 2013; Upton et al., 2016). To advance education, practice and research in the field of nursing, having valid and reliable assessment methods for each RU competence area is crucial (Halm, 2024). To the best of our knowledge, no psychometrically tested instrument has been developed to comprehensively measure nursing students’ RU competence at graduation. Although some related content exists in earlier instruments, none combine and cover the content areas as comprehensively as the CompRU instrument.

## The study

As RU is at the core of evidence-based practice (Belita et al., 2020; Florczak, 2016), there is a need for a more in-depth assessment of nursing students’ RU learning outcomes at graduation and during their transition into working life. An instrument measuring nursing students’ RU competence, based on a knowledge test and self-assessment, could contribute to a more holistic understanding of the effectiveness and quality of nursing education in Europe. Thus, the CompRU instrument, which addresses knowledge of, skills in and attitudes to RU, has been developed for use at the graduation phase of nursing degree programmes (Heikkilä, 2005). The aim of this study was to assess the psychometric properties of CompRU among graduating nursing students in three European countries. The research questions were:

1. What is the internal consistency of the CompRU instrument?
2. What are the measurement properties (item functioning, difficulty level, internal scale validity and discriminatory power) of the Knowledge of RU section?
3. What is the construct validity of the Skills in RU and Attitudes to RU sections?

## Methods

### Design and setting

This cross-sectional study assessed the psychometric properties of the CompRU instrument, focusing on its reliability and validity. Data were collected in three European countries – Finland, Poland and Slovenia – between 2012 and 2017. Although the data are not recent, psychometric properties are generally considered stable over time and therefore not inherently time-dependent.

The countries represent the Northern, Central and Southern regions of Europe. All countries provide bachelor-level nursing education in universities or universities of applied sciences (Heikkilä et al., 2019, 2021) in accordance with European Union directives, with a duration of 3–3.5 years for registered nurse education. Educational institutions offer evidence-based practice and RU content either as separate courses or integrated into other courses and clinical training (Heikkilä et al., 2019, 2021). The Strengthening the Reporting of Observational Studies in Epidemiology (STROBE) checklist (von Elm et al., 2007) was used as a guideline for reporting the study.

### Participants and data collection

The study sample consisted of 648 graduating nursing students who met the inclusion criteria of being full-time or part-time undergraduate nursing students in their final semester. At the national level, each educational institution designated a contact person who collaborated with the national research team members to facilitate student recruitment and coordinate data collection. The response rate was 70% for Finland and 66% for Slovenia, whereas the response rate for Poland could not be determined because complete recruitment data were not available. Students completed either a web-based (Finland) or paper-based (Poland, Slovenia) CompRU questionnaire, and differences in data collection modes may have influenced response completeness across datasets.

The sampling and data collection methods are described in more detail elsewhere (Heikkilä et al., 2019, 2021).

### Instrument

The CompRU instrument was developed 20 years ago to measure nursing students’ research utilisation competence in Finland (Heikkilä, 2005). Its content and concepts, which follow the RU definition used in this study, are still relevant in nursing education across Europe (Dolezel et al., 2021; European Nursing virtual Centre for Learning Evidence-based Practice, 2022). CompRU is based on a literature review and a nationwide thematic enquiry involving 37 principal lecturers from universities of applied sciences (Heikkilä, 2005), and it encompasses essential elements of core RU competencies required for evidence-based practice (Dolezel et al., 2021; European Nursing virtual Centre for Learning Evidence-based Practice, 2022). The instrument’s content validity was assessed in Finland by a group of experts and students involved in nursing education, nurse teacher training and clinical practice in 2005 (*n* = 29), with a subsequent re-evaluation (*n* = 6) conducted in 2012 (Heikkilä, 2005, Heikkilä et al., 2019).

The CompRU instrument (Heikkilä, 2005) consists of 63 items divided into 3 sections: Knowledge of RU, Skills in RU and Attitudes to RU. Knowledge of RU was assessed using a knowledge test comprising 4 multiple-choice questions and 27 matching questions, in which respondents matched concepts to their correct definitions; the list of options exceeded the number of definitions to minimise guessing. Each correct answer was awarded one point; incorrect and ‘I don’t know’ answers received zero points (maximum score: 31). The knowledge test was structured into three main categories and nine subcategories. Self-reported RU skills (16 variables in three categories) were measured using a 5-point Likert scale, ranging from 1 (‘very poor’) to 5 (‘very well’). Self-reported attitudes to RU (16 variables in two categories) were assessed using a 5-point Likert scale, ranging from 1 (‘completely disagree’) to 5 (‘completely agree’) (Table 1).

**Table 1.** Structure of the CompRU instrument (Heikkilä, 2005).

Section (number of items)	Categories (number of items)	Acronym of categories (used in CFA)	Subcategories (number of items)
Knowledge of RU (31)	Acquisition of research knowledge (4)	–	Information sources (2) Methods of information acquisition (2)
	Process of producing research (23)	–	Structure of research papers (4) Research terminology (8) Research approaches (3) Data collection methods (3) Data analysis methods (5)
	Evaluation criteria for research (4)	–	Reliability (3) Clinical relevance (1)
Skills in RU (16)	Acquisition of research knowledge (4)	s_ac	–
	Critical reading of research (8)	s_re	–
	Application of research (4)	s_ap	–
Attitudes to RU (16)	Appreciation of RU (7)	a_ap	–
	Commitment to RU (9)	a_co	–

CompRU: Competence in Research Utilisation; CFA: confirmatory factor analysis; RU: research utilization.

For the international use, the Finnish version of the CompRU instrument was first translated into English and then into Polish and Slovenian using a double-translation procedure (Maneesriwongul and Dixon, 2004). The clarity, comprehensiveness and appropriateness of the translated versions were evaluated by newly graduated nurses in Poland and nursing experts in Slovenia. The translations were found to be linguistically clear and understandable, so no changes were made. However, measurement invariance testing and formal cross-cultural equivalence assessment were not performed.

In earlier studies (Heikkilä et al., 2019, 2021), the internal consistency of the CompRU instrument, measured by Cronbach’s alpha, ranged from 0.78 to 0.90 for the Skills in RU and Attitudes to RU sections, indicating an acceptable level (Tavakol and Dennick, 2011). The preliminary construct validity of the instrument, assessed with principal component analysis (Promax and Kaiser rotation, eigenvalue greater than one), explained 60.6%–63% (Skills in RU) and 46%–51.7% (Attitudes to RU) of the variance in the country-specific data (Heikkilä et al., 2019, 2021), supporting the theoretical structure of the categories. The construct of the RU knowledge test could not be preliminarily tested using principal component analysis because the scale was dichotomous. Therefore, in this study, it was justified to test the measurement properties (item functioning, difficulty level, internal scale validity and discriminatory power) of the knowledge test using Rasch analysis.

### Data analysis

Data were analysed using the SPSS Statistics 29.0 version (IBM Corp., Armonk, NY, USA), Winsteps 5.9.1.0 program with a dichotomous Rasch model and MPlus (Los Angeles, CA, USA) version 7.11 for confirmatory factor analysis (CFA). The number of respondents was large enough for statistical analysis. Analyses were conducted using complete cases only, with no imputation applied. Firstly, descriptive statistics were performed to describe the sample and study variable. In accordance with the theoretical framework, the sum variables were created by calculating mean scores: item responses were summed and divided by the number of items within each scale. Secondly, Cronbach’s alpha values were calculated to measure the internal consistency reliability of the instrument, with an acceptable level of reliability set at ⩾0.70 (Tavakol and Dennick, 2011). Thirdly, item-to-total correlations (*r* ⩾ 0.30) were calculated to describe the item in relation to its theoretical construct (McGahee and Ball, 2009).

Fourthly, a Rasch analysis was performed to evaluate the internal validity of the Knowledge of RU construct using a dichotomous scale. Rating scale functioning was evaluated by inspecting the use of the scale across the items. Internal scale validity was evaluated with item goodness-of-fit statistics, using criteria of infit to MnSq values between 0.7 and 1.4 logits (Bond and Fox, 2007). Unidimensionality of the test was analysed with principal component analysis of the residuals. The first latent variable needed to explain at least 50% of the total variance to support unidimensionality (Bond and Fox, 2007). Person-response validity was assessed using person goodness-of-fit statistics, with infit MnSq values higher than 1.4 logits and *z* values higher than 2.0 (Wright and Linacre, 1994). The percentage of persons outside the recommended goodness-of-fit values was calculated. Up to 5% of the sample could demonstrate misfit; higher percentages were considered to possibly threaten validity. The person-separation reliability index was used to assess if the scale could distinguish persons into at least two groups (Bond and Fox, 2007). A person-item map (Wright map) was used in visual inspection to see how persons and items were distributed along the continuum. Finally, differential item functioning (DIF) was tested between countries to see if the item difficulty calibrations were stable across countries.

Fifthly, CFA was conducted to confirm the construct validity of the sections Skills in RU (16 items) and Attitudes to RU (16 items), which were measured using a five-point Likert scale. The fit of the model was determined by testing the hypothesised model with structural equation modelling and constructed with maximum likelihood estimations. Because sum variables were used in the CFA model, maximum likelihood estimation was considered appropriate. Several criteria were used to examine the goodness-of-fit of the model including non-significant chi-square statistics (χ^2^, *p*, degree of freedom), comparative fit index (CFI), the Tucker–Lewis index (TLI), standardised root mean square residual (SRMR) and root mean square error of approximation (RMSEA). The chi-square test is an absolute test of model fit, in which a probability value (*p*) below 0.05 indicates that the model should be rejected. However, this test is too powerful to reject a model when sample size is large (Babyak and Green, 2010). A normed chi-square (χ^2^/df) was used as an alternative model-fit index to minimise the impact of the sample size, as values <5 are usually considered acceptable (Hooper et al., 2008). For other indices, the CFI takes the model fit into account: it may range between 0 and 1 with values close to 1 indicating very good fit (Bentler, 1990). The CFI value was set in this study at ⩾0.95. Hu and Bentler (1999) recommended a TLI index close to 1.0 and RMSEA values <0.05 (good) to 0.08 (moderate) for a fit model. Furthermore, the SRMR, which is the most sensitive index to detect mis-specified latent structures or factor covariances, was set at ⩽0.08 (Hu and Bentler, 1999).

## Results

### Nursing students’ demographic characteristics

A total of 648 graduating nursing students participated in the study: 40.1% from Finland, 25.9% from Poland and 34% from Slovenia (Table 2).

**Table 2.** Demographic characteristics of nursing students (*n* = 452–648).

Demographic characteristics	*n*	%	Mean	SD	Range
Country	648				
Finland	260	40.1			
Poland	168	25.9			
Slovenia	220	34.0			
Age (years)	641		26.7	6.0	17–58
Gender	644				
Female	570	88.5			
Male	74	11.5			
Prior vocational qualification in healthcare	646				
Yes	257	39.8			
No	389	60.2			
Working experience in healthcare	645				
Yes	342	53.0			
No	303	47.0			
The length of working experience in healthcare (years)	452				
Under 1 year	202	44.7			
1–under 2 years	90	19.9			
2–3 years	39	8.6			
Over 3 years	121	26.8			

SD: standard deviation.

### Internal consistency reliability

Cronbach’s alpha values for internal consistency reliability were 0.881 for Knowledge of RU, 0.905 for Skills in RU and 0.832 for Attitudes to RU, with sum variable ranges of 0.453–0.861, 0.750–0.836 and 0.738–0.799, respectively. The Cronbach’s alpha for the Acquisition of research knowledge category was low (α = 0.453), which may be related to the item formulation and dichotomous scoring. For all sum variables, 75%–100% of the item-to-total correlations met the criterion of *r* ⩾ 0.3, indicating acceptable reliability (McGahee and Ball, 2009) (Table 3).

**Table 3.** Reliability analysis of the CompRU instrument (*n* = 648).

Sections and categories	*n*	Items (*n*)	Cronbach’s alpha	Item-to-total correlation (%, *r* ⩾ 0.30), range
Knowledge of RU^a^	648	31	0.881	(90.3), 0.113–0.608
Acquisition of research knowledge	648	4	0.453	(75), 0.152–0.325
Process of producing research	639	23	0.861	(91.3), 0.167–0.603
Evaluation criteria for research	624	4	0.621	(100), 0.363–0.469
Skills in RU^b^	624	16	0.905	(100), 0.540–0.656
Acquisition of research knowledge	643	4	0.750	(100), 0.443–0.626
Critical reading of research	643	8	0.836	(100), 0.452–0.662
Application of research	640	4	0.823	(100), 0.565–0.731
Attitudes to RU^c^	646	16	0.832	(81.2), 0.061–0.694
Appreciation of RU	646	7	0.799	(100), 0.349–0.628
Commitment to RU	646	9	0.738	(77.8), 0.143–0.610

CompRU: Competence in Research Utilisation; RU: research utilization.

a Knowledge test scoring: one point for correct answer.

b Five-point Likert scale: 1 – very poor; 2 – rather poor; 3 – neither well nor poorly (moderately); 4 – rather well; 5 – very well.

c Five-point Likert scale: 1 – disagree completely; 2 – disagree partially; 3 – neither agree or disagree; 4 – agree partially; 5 – agree completely.

### Measurement properties of Knowledge of RU section based on Rasch analysis

The rating scale of the Knowledge of RU was used as intended. There was monotonic advancement in terms of average measures of each subcategory and the thresholds. Internal scale validity was acceptable when evaluated with goodness-of-fit statistics. In all items, the loadings were within the recommended range. The item MnSq ranged from 1.37 to 0.78 and the *Z*std values from 6.52 to −6.19 (Table 4).

**Table 4.** Item difficulty-based statistics for the Knowledge of RU test (Rasch analysis).

Item number^a^	Subcategory	Measure	SE	Infit MnSq	*Z*std	Outfit MnSq	*Z*std
Q17b	Information sources	1.71	0.14	1.01	0.16	2.55	5.06
Q20c	Research terminology	1.50	0.13	0.91	−1.04	1.17	0.88
Q23a	Data analysis methods	1.43	0.13	1.00	−0.01	1.19	0.98
Q20b	Research terminology	1.13	0.12	0.90	−1.35	0.90	−0.60
Q23e	Data analysis methods	1.02	0.12	1.11	1.60	1.28	1.75
Q24a	Reliability	0.97	0.12	0.87	−2.02	0.75	−1.82
Q20g	Research terminology	0.52	0.11	0.92	−1.48	0.83	−1.53
Q20h	Research terminology	0.55	0.11	1.06	1.10	0.96	−0.29
Q20f	Research terminology	0.45	0.10	0.82	−3.61	0.75	−2.47
Q21a	Research approaches	0.39	0.10	1.07	1.34	1.15	1.37
Q21c	Research approaches	0.39	0.10	1.31	5.35	1.50	4.07
Q18b	Methods of information acquisition	0.30	0.10	1.37	6.52	1.818	6.46
Q23d	Data analysis methods	0.36	0.10	1.31	5.37	1.39	3.31
Q24b	Reliability	0.29	0.10	1.15	2.85	1.26	2.38
Q20e	Research terminology	0.24	0.10	0.78	−4.82	0.67	−3.80
Q17a	Information sources	−0.05	0.10	1.13	2.85	1.40	4.27
Q23c	Data analysis methods	0.03	0.10	0.91	−1.98	0.87	−1.50
Q20a	Research terminology	−0.07	0.10	0.83	−4.07	0.73	−3.55
Q22b	Data collection methods	−0.24	0.10	0.87	−3.10	0.85	−2.01
Q19d	Structure of research papers	−0.38	0.09	0.84	−4.11	0.76	−3.54
Q22a	Data collection methods	−0.40	0.09	1.03	0.70	1.00	−0.04
Q21b	Research approaches	−0.50	0.09	1.23	5.37	1.30	3.95
Q22c	Data collection methods	−0.54	0.09	1.00	0.02	0.93	−1.08
Q23b	Data analysis methods	−0.54	0.09	0.99	−0.16	0.94	−0.95
Q18a	Methods of information acquisition	−0.67	0.09	1.01	0.26	1.04	0.68
Q20d	Research terminology	−0.75	0.09	0.78	−6.19	0.70	−5.25
Q24d	Clinical relevance	−0.73	0.09	0.99	−0.27	1.00	−0.01
Q19a	Structure of research papers	−1.11	0.09	0.93	−2.01	0.92	−1.34
Q19c	Structure of research papers	−1.40	0.09	1.01	0.31	1.09	1.29
Q19b	Structure of research papers	−1.84	0.10	0.92	−1.91	0.81	−2.46
Q24c	Reliability	−2.06	0.10	0.78	−5.09	0.63	−4.46

a The items (questions) are listed in order of difficulty with the most difficult at the top and the easiest at the bottom.

SE: standard error; Infit MnSq: infit mean square; Zstd: standardised *z*-scores; Outfit MnSq: outfit mean square.

The first component explained 44.4% of the variance, suggesting that nearly half of the response variation can be attributed to a single underlying construct, which supports the assumption of unidimensionality (recommended criteria 50%). The largest first contrast component explained 5.1% (eigenvalue 2.24) of the variance. The second, third, fourth and fifth contrasts each explained less than 5% (4.2%, 3.7%, 3.4% and 3.4%, respectively).

**Figure 1.**
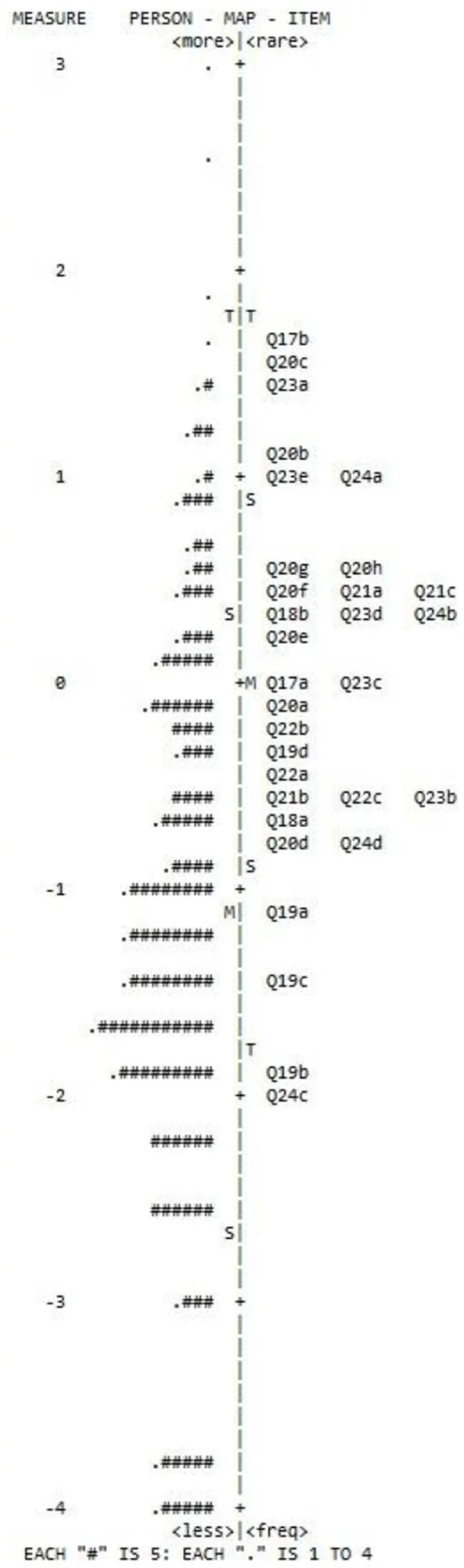
Item-person (Wright) map for the Knowledge of research utilisation test.

Person-response goodness-of-fit values were excellent. Only 0.7% (*n* = 5) of respondents demonstrating misfit to the model. The person separation reliability was 2.20, indicating that the scale could separate respondents into at least two distinct groups (person reliability 0.83). Overall, the items and participants were located predominantly at the same level as the items. However, some participants were located higher than the items indicating that the ability of the participants was higher than the ability reflected by the items. The Wright map of item hierarchy demonstrates a connection of items and participants on a continuum (Figure 1). DIF was identified in 17 items across the countries. DIF was not concentrated in a single subdomain but distributed across the items of the instrument.

### Construct validity of the sections Skills in RU and Attitudes to RU

**Figure 2.**
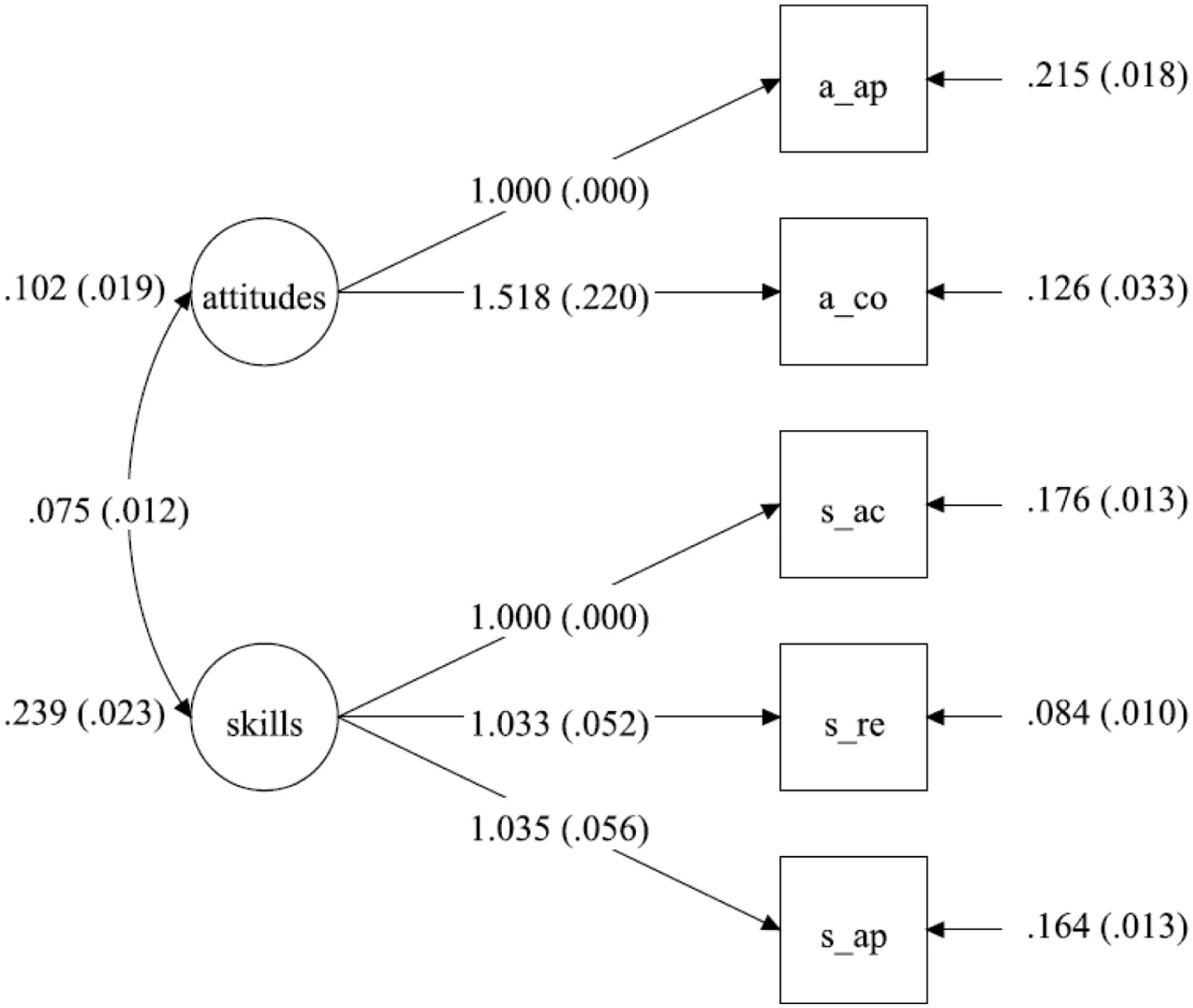
The hypothesised structural model of the CompRU instrument (Skills and Attitudes) as tested by confirmatory factor analysis. CompRU: Competence in Research Utilisation.

CFA was conducted to test the construct validity of the sections Skills in RU and Attitudes to RU. The goodness-of fit indices for the hypothesised model are presented in Figure 2. Within each section, the relations of categories were positive and statistically significant. Latent variables (Skills in RU and Attitudes to RU) were also positively and statistically significantly correlated. Based on the result of the chi-square test, the hypothesised model was not supported (χ^2^ = 14.38, *p* = 0.006). However, a normed chi-square (χ^2^/df = 3.60) gives support for the model. Moreover, other fit parameters (CFI = 0.990, TLI = 0.975 and RMSEA = 0.063, SRMR = 0.016) confirm the goodness-of-fit for the hypothesised model (Table 5).

**Table 5.** Goodness-of-fit indices for the hypothesised model of the sections research utilisation skills and attitudes to research utilisation.

χ^2^	df	χ^2^/df	*p*-Value	CFI	TLI	SRMR	RMSEA (90% Cl)	*p* ⩽0.05	AIC	BIC
14.38	4	3.60	0.006	0.990	0.975	0.016	0.063 (0.030–0.100)	0.223	4898	4970

CFI: comparative fit index; TLI: Tucker–Lewis index; SRMR: standardised root mean square residual; RMSEA: root mean square error of approximation; AIC: Akaike Information Criterion; BIC: Bayesian Information Criterion.

## Discussion

This study highlights the role of RU as a fundamental driver of evidence-based practice, the ultimate goal of RU. Deficiencies in RU competence among nurses constitute a significant obstacle to evidence-based practice (Ferreira et al., 2021; Hines et al., 2022). Assessment is needed to determine whether competence in RU is achieved through nursing education in Europe, as European Union directives require, and to provide knowledge that informs the development of nursing education. The aim of this study was to assess the psychometric properties of the CompRU instrument in the context of graduating nursing students using data from three European countries. The CompRU instrument, which addresses knowledge of, skills in and attitudes to RU, has been developed for use at the graduation phase of nursing degree programmes (Heikkilä, 2005).

The Cronbach’s alpha coefficient, which measured the internal consistency reliability of the CompRU instrument, was at an acceptable level (α ⩾ 0.70; Tavakol and Dennick, 2011), despite two categories within the Knowledge of RU section showing lower consistency. In the category Acquisition of research knowledge, the formulation of the questions (multiple-choice questions) differed from that in other categories (matching questions), which may partly explain the observed results. Additionally, the dichotomous scoring format may have contributed to the low reliability estimates, although Cronbach’s alpha is an appropriate reliability coefficient for dichotomously scored items because it is equivalent to the Kuder–Richardson Formula 20 (KR-20; Madadizadeh and Bahariniya, 2025). The category comprised four items that address closely related aspects, namely sources of information and methods of obtaining information, which could have also contributed to the findings. Nevertheless, it was justified to include multiple questions on these topics to adequately capture respondents’ understanding of how research knowledge is acquired, especially since a low number of questions can lead to a lower value of Cronbach’s alpha (Tavakol and Dennick, 2011). Furthermore, these issues were also addressed in the Skills in RU section, where the internal consistency was acceptable (Cronbach’s α = 0.750), supporting the inclusion of more questions. Across all sections and categories, 75%–100% of the item-to-total correlations, which indicate how well each item relates to its theoretical construct, met the criterion (*r* ⩾ 0.30; McGahee and Ball, 2009). This demonstrated good consistency among the items, with no items needing to be removed.

A Rasch analysis, which has been used only to a limited extent in nursing science (Stolt et al., 2022), was conducted to examine the difficulty level and discriminatory power of the 31 dichotomous items in the test measuring knowledge of RU. Internal scale validity was supported in terms of item fit; however, regarding unidimensionality, the first component explained only 44.4% of the total variance. Similarly, the proportion of variance explained by the second dimension (first contrast component) was slightly higher – 5.1% – as recommended. The low unidimensionality may be explained by the possible multidimensionality in the RU knowledge section. There could be overlapping items or heterogeneous item content, as items may vary in format, difficulty, or cognitive demand. However, item fit was satisfactory among all items, and no misfitting items were detected. Furthermore, person separation reliability was satisfactory, indicating the ability to distinguish between respondents with more knowledge and those with less. DIF was identified in 17 items across the 3 countries, suggesting that some items may function differently across cultural contexts and potentially affect the comparability of cross-country scores, as differences could partly reflect measurement bias rather than true variation. This could potentially be related to differences in educational systems and approaches to teaching RU (Skela-Savič et al., 2020). However, cross-country comparisons remain broadly interpretable, albeit with caution. Further refinement of these items is recommended, including potential rewording and improved cultural adaptation. In addition, future work could benefit from international expert evaluation to identify overlapping or redundant items in the RU Knowledge test, as well as analyses of cross-cultural semantic equivalence to ensure consistent item meaning across countries.

The construct validity of the Skills in RU and Attitudes to RU sections was assessed with CFA. The relationships between the categories within the Skills and Attitudes sections, as well as between the two sections, were positive and statistically significant, supporting the assumption that the CompRU instrument measured the integration of skills in RU and attitudes to RU in this dataset. Although the chi-square test, which is sensitive to large sample sizes (Babyak and Green, 2010), did not support construct validity, the normed chi-square and other fit indices demonstrated adequate model fit (Bentler, 1990; Hu and Bentler, 1999). The SRMR as a goodness-of-fit measure was very satisfactory indicating there is minimal threat to model misspecification. It allows assessing the average magnitude of the discrepancies between observed and expected correlations as an absolute measure of (model) fit criterion (Hu and Bentler, 1999). Thus, the hypothetical model functioned as intended, and no further modifications were necessary. Furthermore, since no formal cross-cultural equivalence or measurement invariance testing was conducted in this study, it is recommended that such testing be carried out in future international validation studies.

Although the CompRU instrument was developed 20 years ago to assess competence in RU among graduating nursing students in Finland (Heikkilä, 2005), its content and underlying conceptual framework remain relevant in contemporary higher nursing and healthcare education across Europe (Dolezel et al., 2021; European Nursing Virtual Centre for Learning Evidence-Based Practice, 2022), including the Nordic countries (Gonzalez et al., 2024; Immonen et al., 2025). Because self-assessment methods can be inaccurate (González-Betancor et al., 2019), they are recommended to be supplemented with objective assessments, such as knowledge tests (Hagedorn Wonder and Spurlock, 2020). Psychometric testing indicates that the CompRU instrument, which includes both a knowledge test and a self-assessment of skills and attitudes, is a promising tool for measuring RU competence. Instruments that capture all of these attributes are essential, given that knowledge, skills and attitudes are inseparably intertwined in the formation of competence (Belita et al., 2020). In the future, it is recommended to more closely examine the difficulty level and sensitivity of the CompRU instrument in the RU Knowledge section. Ensuring the semantic equivalence and sensitivity of the different language versions of the CompRU instrument would also be an important area for future research.

The content and underlying concepts of the CompRU instrument are broadly applicable to RU. In addition to being used among graduating nursing students, the CompRU instrument has also been applied to assess the RU competence of professional nurses (Salminen et al., 2022) and postgraduate nursing students (Heikkilä et al., 2024). In these studies, internal consistency has aligned with the findings of the present study, and construct validity has been consistent with studies involving graduating nursing students (Heikkilä et al., 2019, 2021). Although the CompRU instrument is primarily designed for undergraduate nursing students, it may be applicable among professional nurses, postgraduate nursing students and – with minor adaptations (e.g. replacing ‘nurse’ with the relevant professional title) – other healthcare professionals. However, more research is needed.

### Strengths and limitations

The study has several strengths and limitations. Firstly, the study included a large, international dataset from three European countries. However, the samples were not nationally representative and may not fully reflect the overall situation of RU education and competence in each country. Consequently, the findings should be interpreted as reflecting the participating student groups in each country rather than allowing national-level generalisations. Secondly, a strength is that the psychometric testing was conducted using both classical test theory and modern test theory. Thirdly, although the psychometric testing indicates that the CompRU instrument is a promising tool for measuring RU competence, some instabilities were observed – likely attributable to a minor translation and technical error in the Polish version of the questionnaire (Heikkilä et al., 2019) – despite standardised translation procedures. These issues are unlikely to have substantially influenced the overall results. Furthermore, although European reforms in nursing education have aimed at improving the uniformity and quality of nursing education to ensure generic professional competence (Directive 2005/36/EC; Directive 2013/55/EU), differences in educational systems, curriculum and RU teaching content may have influenced the findings of this study. Also, although the data were collected between 2012 and 2017, psychometric properties are not inherently time-dependent. The theoretical foundations of RU and evidence-based practice have remained stable over the past two decades and recent contextual changes (e.g. the COVID-19 pandemic and the increasing use of technology in nursing education) are not expected to markedly alter competencies related to acquiring, reading, appraising and applying research. Therefore, the instrument’s psychometric validity is not compromised. Future studies may consider re-examining the CompRU instrument using more recent data to assess its performance across evolving educational contexts.

## Conclusion

Psychometric testing conducted on a sample of 648 nursing students graduating from 3 European countries showed that the CompRU instrument is a potentially valuable tool for assessing nursing students’ RU competence at the point of graduation in nursing degree programmes. Based on both classical and modern test theory approaches, the instrument demonstrates acceptable psychometric properties, although some items could benefit from revisions. In the RU Knowledge test, item content could be reviewed to identify potential overlap or redundancy and to ensure cross-cultural equivalence, so that item meaning is consistent across countries. Given the absence of any psychometrically tested instrument that captures nursing students’ RU competence in a comprehensive manner at graduation, the CompRU instrument fills an important gap in nursing education and research. Future research should further examine the characteristics of the CompRU instrument, particularly the difficulty level and sensitivity of the RU Knowledge section, among nursing students and other groups of healthcare professionals, ideally including international samples from multiple European countries.

**Key points for policy, practice and/or research**

- Evidence-based practice is an internationally accepted standard in healthcare, underpinned by RU as a key driver.
- The CompRU instrument is a promising tool for measuring nursing students’ RU competence at graduation and during their transition into working life.
- An instrument with documented psychometric properties enables nurse educators and researchers to evaluate whether nursing education meets the RU competence requirements outlined in European Union directives.
- Given the current absence of any psychometrically tested instrument that captures nursing students’ RU competence in a comprehensive manner at graduation, the CompRU instrument fills an important gap in nursing education and research.
- The CompRU instrument may also be applicable among professional nurses, postgraduate nursing students and – with minor adaptations (e.g. replacing ‘nurse’ with the relevant professional title) – other healthcare professionals.

## References

[bibr1-17449871261463995] ALLEA, All European Academies (2023) The European code of conduct for research integrity – Revised edition 2023. Berlin. Available at: https://allea.org/wp-content/uploads/2023/06/European-Code-of-Conduct-Revised-Edition-2023.pdf (accessed 22 December 2025). DOI: 10.26356/ECOC.

[bibr2-17449871261463995] Babyak MA Green SB (2010) Confirmatory factor analysis: An introduction for psychosomatic medicine researchers. Psychosomatic Medicine 72: 587–597. DOI: 10.1097/PSY.0b013e3181de3f8a 20467001

[bibr3-17449871261463995] Belita E Squires JE Yost J , et al. (2020) Measures of evidence-informed decision-making competence attributes: A psychometric systematic review. BMC Nursing 19: 44. DOI: 10.1186/s12912-020-00436-8. 32514242 PMC7254762

[bibr4-17449871261463995] Bentler PM (1990) Comparative fit indexes in structural models. Psychological Bulletin 107(2): 238–246. DOI: 10.1037/0033-2909.107.2.238 2320703

[bibr5-17449871261463995] Bond TG Fox CM (2007) Applying the Rasch Model: Fundamental Measurement in the Human Sciences, 3rd edn. New York, NY: Routledge.

[bibr6-17449871261463995] Costa ICP Mendes KDS Freitas PS (2025) Literature search strategies: A guideline for identifying the best evidence in healthcare. Texto & Contexto Enfermagem 34, e20230405. DOI: 10.1590/1980-265X-TCE-2023-0405en

[bibr7-17449871261463995] Directive 2005/36/EC. The European Parliament and the Council. The recognition of professional qualifications. Available at: http://data.europa.eu/eli/dir/2005/36/oj (accessed 22 December 2025).

[bibr8-17449871261463995] Directive 2013/55/EU. The European Parliament and the Council. Amendment of the Directive 2005/36/EC. Available at: http://data.europa.eu/eli/dir/2013/55/oj (accessed 22 December 2025).

[bibr9-17449871261463995] Dolezel J Zelenikova R Finotto S , et al. (2021) Core evidence-based practice competencies and learning outcomes for European nurses: Consensus statements. Worldviews on Evidence-Based Nursing 18: 226–233. DOI: 10.1111/wvn.12506. 34031973 PMC8251814

[bibr10-17449871261463995] Estabrooks CA (1999) The conceptual structure of research utilization. Research in Nursing & Health 22: 203–216. 10344701 10.1002/(sici)1098-240x(199906)22:3<203::aid-nur3>3.0.co;2-9

[bibr11-17449871261463995] Estabrooks CA Squires JE Strandberg E , et al. (2011) Towards better measures of research utilization: A collaborative study in Canada and Sweden. Journal of Advanced Nursing 67: 1705–1718. DOI: 10.1111/j.1365-2648.2011.05610.x. 21477116

[bibr12-17449871261463995] European Nursing virtual Centre for Learning Evidence-based Practice (ENCLEBP) (2022) Guidelines for teaching and learning evidence-based practice in the European nursing curriculum. EBP e-Toolkit Project. Available at: https://europeannursingebp.eu/guidelines/ (accessed 4 January 2026).

[bibr13-17449871261463995] Ferreira MBG Dos Santos FMM de Souza Lopes RA , et al. (2021) Barriers to research utilization influencing patient safety climate: A cross-sectional study. International Journal of Nursing Practice 27: e12959. DOI: 10.1111/ijn.12959. 33977599

[bibr14-17449871261463995] Florczak KL (2016) Evidence-based practice: What’s new is old. Nursing Science Quarterly 29: 108–112. DOI: 10.1177/0894318416630096. 26980886

[bibr15-17449871261463995] Gonzalez MT Nilsen Bakken L Horntvedt M-ET , et al. (2024) Norwegian nurse educators’ self-rating of competencies: A nationwide cross-sectional web-survey. International Journal of Nursing Education Scholarship 21: 20230040. DOI: 10.1515/ijnes-2023-0040. 38563612

[bibr16-17449871261463995] González-Betancor SM Bolívar-Cruz A Verano-Tacoronte D (2019) Self-assessment accuracy in higher education: The influence of gender and performance of university students. Active Learning in Higher Education 20: 101–114. DOI: 10.1177/1469787417735604.

[bibr17-17449871261463995] Hagedorn Wonder A Spurlock D Jr (2020) A national study across levels of nursing education: Can nurses and nursing students accurately estimate their knowledge of evidence-based practice? Nursing Education Perspectives 41: 77–82. DOI: 10.1097/01.NEP.0000000000000528. 31232879

[bibr18-17449871261463995] Halm M (2024) A compendium of evidence-based practice instruments for nursing education, practice and research. Worldviews on Evidence-Based Nursing 21: 6–13. DOI: 10.1111/wvn.12703 38197468

[bibr19-17449871261463995] Heikkilä A (2005) Research knowledge utilisation of polytechnic nursing students on graduation. PhD Thesis [original text in Finnish, abstract in English], University of Turku, Finland.

[bibr20-17449871261463995] Heikkilä A Kaučič BM Filej B , et al. (2021) Slovenian nursing students’ Competence in Research Utilization, and the support they received during clinical practice. Central European Journal of Nursing and Midwifery 12: 376–384. DOI: 10.15452/CEJNM.2021.12.0013.

[bibr21-17449871261463995] Heikkilä A Kwiecień-Jaguś K Hupli M , et al. (2019) Polish and Finnish nursing students’ attitudes, knowledge and skills related to research utilisation. Medycyna Ogólna i Nauki o Zdrowiu 25: 181–186. DOI: 10.26444/monz/111726.

[bibr22-17449871261463995] Heikkilä A Salminen L Hupli M , et al. (2024). Research utilization competence of postgraduate nursing students: A cross-sectional study. Nordic Journal of Nursing Research 44: 1–8. DOI: 10.1177/20571585241234607.

[bibr23-17449871261463995] Hines S Ramsbotham J Coyer F (2022) Registered nurses’ experiences of reading and using research for work and education: A qualitative research study. BMC Nursing 21: 114. DOI: 10.1186/s12912-022-00877-3. 35549936 PMC9096067

[bibr24-17449871261463995] Hooper D Coughlan J Mullen M (2008) Structural equation modelling: Guidelines for determining model fit. The Electronic Journal of Business Research Methods 6: 53–60.

[bibr25-17449871261463995] Hu L Bentler PM (1999) Cutoff criteria for fit indexes in covariance structure analysis: Conventional criteria versus new alternatives. Structural Equation Modeling: A Multidisciplinary Journal 6: 1–55. DOI: 10.1080/10705519909540118.

[bibr26-17449871261463995] Hu W-L Hong Y Wang H , et al. (2024) Assessing evidence-based practice competencies of undergraduate nursing students using a developed multi-criteria decision-analysis model. Nurse Education in Practice 76: 103919. 10.1016/j.nepr.2024.103919 38387278

[bibr27-17449871261463995] Immonen K Tuomikoski A-M Mikkonen K , et al. (2025) Evidence-based healthcare competence of social- and healthcare educators: A cross-sectional study. Journal of Advanced Nursing 81: 300–315. DOI: 10.1111/jan.16230. 38733079 PMC11638502

[bibr28-17449871261463995] Madadizadeh F Bahariniya S (2025) Tutorial on internal consistency assessment by Cronbach’s alpha and McDonald’s omega. Perioperative Care and Operating Room Management 41: 100568. DOI: 10.1016/j.pcorm.2025.100568.

[bibr29-17449871261463995] Maneesriwongul W Dixon JK (2004) Instrument translation process: A methods review. Journal of Advanced Nursing 48: 175–186. DOI: 10.1111/j.1365-2648.2004.03185.x. 15369498

[bibr30-17449871261463995] McGahee T Ball J (2009) How to read and really use an item analysis. Nurse Educator 34: 166–171. DOI: 10.1097/NNE.0b013e3181aaba94. 19574855

[bibr31-17449871261463995] Mrayyan MT Abunab HY Abu Khait A , et al. (2023) Competency in nursing practice: A concept analysis. BMJ Open 13: e067352. doi:10.1136/bmjopen-2022-067352 PMC10255110 37263688

[bibr32-17449871261463995] Patelarou A Schetaki S Giakoumidakis K , et al. (2021) Validation of the Evidence-Based Practice Competence Questionnaire for nursing students: A cross-sectional study in Greece. Nursing Reports 11: 765–774. DOI: 10.3390/nursrep11040073. 34968267 PMC8715452

[bibr33-17449871261463995] Price B (2015) Understanding attitudes and their effects on nursing practice. Nursing Standard 30: 50–57. DOI: 10.7748/ns.30.15.50.s51. 26647707

[bibr34-17449871261463995] Qiu C Feng X Reinhardt JD , et al. (2019) Development and psychometric testing of the Research Competency Scale for Nursing Students: An instrument design study. Nurse Education Today 79: 198–203. DOI: 10.1016/j.nedt.2019.05.039. 31154266

[bibr35-17449871261463995] Ruzafa-Martinez M Lopez-Iborra L Moreno-Casbas T , et al. (2013) Development and validation of the competence in evidence-based practice questionnaire (EBP-COQ) among nursing students. BMC Medical Education 13: 19. DOI: 10.1186/1472-6920-13–19. 23391040 10.1186/1472-6920-13-19 PMC3598337

[bibr36-17449871261463995] Salminen L Kaučič BM Filej B , et al. (2022) Slovenian nurses’ research utilization competence and received support from nurse managers. Pielegniarstwo XXI wieku / Nursing in the 21st Century 21: 5–11. DOI: 10.2478/pielxxiw-2022-0002.

[bibr37-17449871261463995] Skela-Savič B Gotlib J Panczyk M , et al. (2020) Teaching evidence-based practice (EBP) in nursing curricula in six European countries – A descriptive study. Nurse Education Today 94: 104561. 32905986 10.1016/j.nedt.2020.104561

[bibr38-17449871261463995] Stolt M Kottorp A Suhonen R (2022) The use and quality of reporting of Rasch analysis in nursing research: A methodological scoping review. International Journal of Nursing Studies 132: 104–244. DOI: 10.1016/j.ijnurstu.2022.104244. 35635906

[bibr39-17449871261463995] Strandberg E Eldh AC Forsman H , et al. (2014) The concept of research utilization as understood by Swedish nurses: Demarcations of instrumental, conceptual, and persuasive research utilization. Worldviews on Evidence-Based Nursing 11: 55–64. DOI: 10.1111/wvn.12013. 23879321

[bibr40-17449871261463995] Tavakol M Dennick R (2011) Making sense of Cronbach’s alpha. International Journal of Medical Education 2: 53–55. DOI: 10.5116/ijme.4dfb.8dfd. 28029643 PMC4205511

[bibr41-17449871261463995] Unal A Teskereci G (2022) Mapping the evidence-based practice research field in nursing from 1995 to 2021: A bibliometric analysis. International Journal of Nursing Knowledge 33: 196–206. DOI: 10.1111/2047-3095.12347. 34693654

[bibr42-17449871261463995] Upton P Scurlock-Evans L Upton D (2016) Development of the Student Evidence-based Practice Questionnaire (S-EBPQ). Nurse Education Today 37: 38–44. DOI: 10.1016/j.nedt.2015.11.010. 26627595

[bibr43-17449871261463995] Vitello S Greatorex J Shaw S (2021) What is Competence? A Shared Interpretation of Competence to Support Teaching, Learning and Assessment. Cambridge University Press & Assessment.

[bibr44-17449871261463995] Von Elm E Altman DG Egger M , et al. (2007) The strengthening the reporting of observational studies in epidemiology (STROBE) statement: Guidelines for reporting observational studies. The Lancet 370(9596): 1453–1457. 10.1016/S0140-6736(07)61602-X 18064739

[bibr45-17449871261463995] World Health Organization (2016) Nurse educator core competencies. Available at: https://www.who.int/publications/b/33494 (accessed 6 January 2026).

[bibr46-17449871261463995] Wright B Linacre J (1994) Reasonable mean-square fit statistics. Rasch Measurement Transactions 8: 370.

[bibr47-17449871261463995] Xia Y Huang H Halili X , et al. (2023) Instruments for measuring nursing research competence: A COSMIN‑based scoping review. BMC Nursing 22: 410. DOI: 10.1186/s12912-023-01572-7. 37907869 PMC10617091

